# PERK: a novel therapeutic target for neurodegenerative diseases?

**DOI:** 10.1186/alzrt260

**Published:** 2014-05-29

**Authors:** Tao Ma, Eric Klann

**Affiliations:** 1 Center for Neural Science, New York University, 4 Washington Place, Room 809, New York, NY 10003, USA

## Abstract

Identification of therapeutic targets based on novel mechanistic studies is urgently needed for neurodegenerative diseases such as Alzheimer’s disease, Parkinson’s disease, and prion disease. Multiple lines of evidence have emerged to suggest that inhibition of the stress-induced endoplasmic reticulum kinase PERK (protein kinase RNA-like endoplasmic reticulum kinase) is a potential therapeutic strategy for these diseases. A recently published study demonstrated that oral treatment with a newly characterized PERK inhibitor was able to rescue disease phenotypes displayed in prion disease model mice. Here, we discuss the background and rationale for targeting PERK as a viable therapeutic approach as well as implications of these findings for other neurodegenerative diseases. The promise and caveats of applying this strategy for disease therapy also are discussed.

## Introduction

Diseases such as Alzheimer’s disease (AD), Huntington’s disease (HD), Parkinson’s disease (PD), and prion disease usually are classified as neurodegenerative diseases that represent a heterogeneous group of disorders characterized by progressive structural or functional neuronal loss or both. Neurodegenerative diseases present great challenges for human public health because of the prevalence of the diseases, the severity of clinical symptoms (usually lethal in the end), and the lack of effective treatments. Great efforts have been made to try to understand the etiology of neurodegenerative diseases. At the subcellular and molecular levels, these diseases appear to share many key similarities, which are potential mechanisms underlying the pathophysiology and thus are appealing as therapeutic targets. In a recently published study by Moreno and colleagues [1], oral treatment of a newly characterized and specific inhibitor of the endoplasmic reticulum-localized kinase PERK (protein kinase RNA-like endoplasmic reticulum kinase), termed GSK2606414 [2], was demonstrated to rescue clinical symptoms as well as brain pathology in a mouse model of prion disease. Together with other recent relevant studies, these findings have identified a potential novel therapeutic avenue for drug development to treat neurodegenerative diseases.

## PERK activation during stress response: protective or detrimental?

A common feature among neurodegenerative diseases is the accumulation of misfolded proteins, which constitutes significant cellular stress that triggers the activation of multiple signaling cascades and the unfolded protein response (UPR). The UPR is mediated through three key effectors: inositol-requiring enzyme 1, activating transcription factor 6, and PERK [3]. As a crucial component of the UPR, PERK activation results in phosphorylation and inhibition of eukaryotic initiation factor 2α (eIF2α), a translational factor that controls the initiation step of protein synthesis which has been implicated in long-term synaptic plasticity and memory formation [4]. As a result of increased phosphorylation of eIF2α, global protein synthesis would be inhibited as a component of the cellular stress response [5]. Short-term reduction of general protein synthesis in response to cellular stress typically is considered to be protective because it allows cells to conserve energy resources while enhancing the gene-specific translation of stress-related mRNAs, thereby reconfiguring gene expression to cope with the cellular stress [6]. In contrast, during severe and prolonged forms of cellular stress that are associated with pathological conditions such as are found in neurodegenerative diseases, PERK activation is protracted such that eIF2α is abnormally hyperphosphorylated for long periods of time, leading to a long-lasting repression of mRNA translation. An enhanced and prolonged increase in eIF2α phosphorylation inevitably would be detrimental for cognitive function because intact mechanisms are necessary to trigger *de novo* protein synthesis which are required for long-lasting neuronal plasticity and memory consolidation [7,8]. In short, a balanced UPR/PERK/eIF2α pathway is critical for maintaining cellular homeostasis during stress conditions.

## PERK suppression as a strategy for neurodegenerative diseases: promises and caveats

In the study by Moreno and colleagues, prion-infected mice were orally treated with GSK2606414 either before or after the occurrence of clinical features of prion disease, and remarkably the treatments successfully prevented or rescued both disease-related behavioral defects (mostly) and several brain pathologies. Importantly, the therapeutic effects of the GSK2606414 treatment correlated well with the restoration of both general protein synthesis and the levels of several synaptic proteins in the hippocampus [1]. GSK2606414 appears to be ideal from a ‘translational’ perspective because of its high selectivity and potency for PERK compared with other kinases and has excellent blood–brain barrier penetration. Given that dysregulation of the PERK/eIF2α pathways likely represents a common crucial pathological feature in neurodegenerative diseases [9] and has been well linked to cognitive impairments (as discussed above), determining whether GSK2606414 is effective in other disease models such as AD, PD, or HD is important. In support of this idea, we recently demonstrated that genetic knockdown of PERK post-development in the forebrain of AD model mice was able to prevent AD-associated synaptic plasticity failure and memory impairments [10]. Furthermore, using a different small-molecule PERK inhibitor, we have observed that β-amyloid-induced hippocampal long-term potentiation failure is reversed by PERK inhibition (unpublished data).

On the other hand, it is important to be aware of the caveats in proposing PERK inhibitors as a potential therapy for neurodegenerative diseases. First, excessive repression of PERK, as a kinase involved in a generic cellular stress-response pathway, could hamper the ability of neurons to respond to cellular stress and could disrupt normal regulation of protein synthesis, thereby causing synaptic dysfunction. For example, it was shown that brain-specific knockdown of PERK in mice resulted in dramatic impairments in behavioral flexibility in multiple cognitive tasks [11]. Therefore, fine-tuning the dose and duration of treatment with any PERK inhibitor will be critical to achieve the desired outcomes: re-establishing the capability of triggering *de novo* protein synthesis in response to learning while retaining the cellular stress-response component of the UPR-PERK-eIF2α pathway. Second, in addition to its critical role in the brain, PERK activity is essential for skeletal system development and normal pancreatic function [12]. Indeed, Wolcott-Rallison syndrome in humans, which is characterized by early-onset diabetes mellitus, has been linked to mutations in the *EIF2AK3* gene in humans [13]. In agreement, it is not surprising that Moreno and colleagues reported that GSK2606414 caused weight loss and hyperglycemia in mice. So that these undesired and harmful side effects can be avoided, it would be ideal to modify the inhibitors so that they would act predominantly in the brain without effects in peripheral tissues and organs. Third and last, the study by Moreno and colleagues also found that memory loss in prion disease model mice as evaluated by performance in the object recognition task was not restored if the PERK inhibitor treatment was started at a relatively late stage, after the onset of neuronal loss and the appearance of clinical symptoms of the disease. These findings reinforce the importance of early intervention for neurodegenerative disease therapies, which would require the development of appropriate biomarkers to detect these diseases before the manifestation of clinical symptoms [14].

## Conclusions

Drug development for neurodegenerative diseases such as AD has been unsuccessful [15], and thus identification of alternative therapeutic targets based on novel mechanistic studies is urgently needed. Dysregulation of the UPR/PERK/eIF2α signaling pathway now has been identified as a potential contributing factor to pathophysiology associated with neurodegenerative diseases, and PERK has emerged as a potential novel therapeutic target. The findings of Moreno and colleagues support the aforementioned ideas by demonstrating the ameliorating effects of a novel small-molecule PERK inhibitor on disease phenotypes displayed by prion disease model mice. It will be exciting to determine whether PERK inhibitors can also alleviate the pathophysiology observed in other mouse models of neurodegenerative diseases and for PERK inhibitors to be tested in clinical settings as therapies for humans with neurodegenerative disease. At the same time, we should be cautious about applying this therapeutic strategy and be mindful not to disrupt the cellular and synaptic homeostasis by excessive repression of PERK-eIF2α signaling.

## Abbreviations

AD: Alzheimer’s disease; eIF2α: Eukaryotic initiation factor 2α; HD: Huntington’s disease; PD: Parkinson’s disease; PERK: protein kinase RNA-like endoplasmic reticulum kinase; UPR: Unfolded protein response.

## Competing interests

The authors declare that they have no competing interests.
